# Surgical outcomes and complications of posterior hemivertebra resection in children younger than 5 years old

**DOI:** 10.1186/s13018-016-0381-2

**Published:** 2016-04-25

**Authors:** Jianwei Guo, Jianguo Zhang, Shengru Wang, Yanbin Zhang, Yang Yang, Xinyu Yang, Lijuan Zhao

**Affiliations:** Department of Orthopedics, Peking Union Medical College Hospital, 1 Shuai Fu Yuan, Beijing, 100730 People’s Republic of China

**Keywords:** Congenital scoliosis, Surgical outcome, Hemivertebra resection, Posterior surgery, Complication

## Abstract

**Background:**

There have been many reports on posterior hemivertebra resection. However, there were few articles in very young cases. This is a clinical retrospective study to evaluate the complications and efficacy of posterior hemivertebra resection in very young cases.

**Methods:**

From January 2003 to January 2012, 39 consecutive cases of congenital scoliosis with hemivertebra were retrospectively investigated in our hospital, including 18 females and 21 males, aged from 21 to 65 months old (average 42.4 months). All the cases underwent posterior hemivertebra resection with transpedicular instrumentation. Clinical charts and radiographs of spine were retrospectively reviewed to record complications and outcomes postoperatively and at the latest follow-up.

**Results:**

Mean operation time was 186.4 min (90–280 min) with average blood loss of 306.6 (100–700) ml. The total number of hemivertebrae resected was 43, including one hemivertebra in 35 cases and two hemivertebrae in 4 cases. The mean number of fused segments was 3.4, including 22 cases (56.4 %) with bisegmental fusion and 4 cases with mesh cage. Average follow-up period was 5.4 (3 to 11) years. There was a mean improvement rate of 83.6 % in the segmental scoliosis from 38.4° before surgery to 6.3° after operation and a mean improvement rate of 81.9 % in segmental kyphosis from 17.1° to 3.2° over the same time. The spontaneous correction rates of the compensatory cranial curve and compensatory caudal curve were 65.7 and 66.9 %, respectively. There were three complications (two cases),one pedicle fractures, one rod breakages, and one additional surgery for curve progression. Besides these, two additional surgeries (two cases) were performed to remove the instrumentation for pedicle elongation in the follow-up. There was no neurological complication.

**Conclusions:**

Posterior hemivertebra resection with transpedicular instrumentation is a safe and effective procedure in very young congenital scoliosis cases. Earlier surgeries can achieve short fusion and save more mobile segments. However, complications associated with implants and spinal growth still remain major concerns.

## Background

As a kind of failure of vertebral formation, hemivertebra is the most frequent cause of congenital scoliosis [1]. Hemivertebrae, except for incarcerated, unsegmented, or balanced hemivertebra, have great growth potential similar to normal vertebrae and create a rigid and wedge-shaped deformity during spinal growing period. The curve progression and the ultimate severity of the curve depends on the type of hemivertebrae, the location, the number of hemivertebrae, and their relationship with each other [2]. Besides local deformity, the compensatory curve will progress and become structural. Long fusion will be needed and has to include the rigid structural compensatory curve in adolescent period. Considering the poor natural history of hemivertebra, early surgical treatment is required in most cases [1, 3–6].

Since Royle first described hemivertebra resection, several surgical procedures have been introduced to deal with this kind of deformity [7–25]. Compared with one-stage or two-stage anterior and posterior hemivertebra resection, posterior hemivertebra resection has been proven to be a safe and effective procedure for congenital scoliosis [26]. However, the surgical outcome with a long follow-up is still the biggest challenge for posterior hemivertebra resection in very young children. The purpose of this study is to evaluate surgical outcomes of posterior hemivertebra resection with transpedicular instrumentation with average 5.4 years’ follow-up and analyze complications following posterior hemivertebra resection in children younger than 5 years old.

## Methods

Thirty-nine consecutive patients with non-incarcerated hemivertebra were treated by posterior hemivertebra resection in our hospital from January 2003 to January 2012. The patients included 18 females and 21 males, aged from 21 to 65 months old (average 42.4 months). Of the 39 patients, 2 cases had congenital cardiac malformation, 1 case had release surgery of tethered cord syndrome (TCS) before, and 1 case had syringomyelia. All the patients were performed by the corresponding author. And this study was approved by the Ethics Committee of Peking Union Medical College Hospital (see the ethic approval in the supplementary material).

Before surgery, a complete physical examination and radiographic evaluation were performed for occult congenital anomalies, including standing long cassette anterior–posterior (AP) and lateral (L) radiographs, spine CT, and spinal MRI. Of the 39 patients, 35 patients had only one hemivertebra and 4 cases had two hemivertebrae. The locations of hemivertebrae were identified by radiographs, including 8 in the upper thoracic spine (T1-T5), 5 in the main thoracic spine (T6-T9), 20 in the thoralumbar (T10-L2), and 10 in the lumbar and lumbosacral spine (L3-S1).

Radiographic parameters, including Cobb’s angles in the coronal and sagittal planes, coronal balance (CB), and sagittal balance (SB), were measured in standing AP X-ray examination of the whole spine preoperatively, postoperatively, and at the latest follow-up. The method of measurements was described by Ruf and Harms in 2002 [13]. Operative reports were reviewed to obtain the operative time, bleeding volume, and intraoperative complications. Medical records were reviewed to identify complications in the peri-operative and follow-up periods.

### Operative technique

**Fig. 1:**
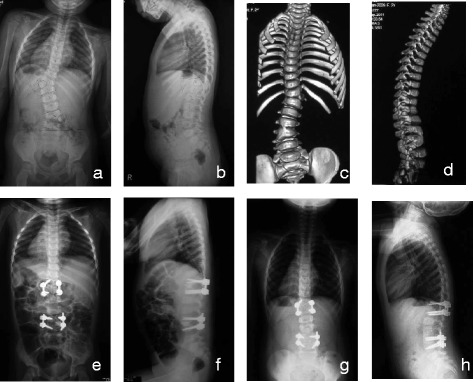
An infant with congenital scoliosis. Preoperative radiographs (**a**, **b**) and CT 3D reconstruction (**c**, **d**) showed fully segmented hemivertebra of T12/L1 and semi-segmented hemivertebra of L4/L5 with coronal segmental scoliosis of 40° and 32° and segmental kyphosis of 32°. Post-operational radiographs (**e**, **f**) showed T12/L1 and L4/L5 hemivertebra resection and internal fixation, and the scoliosis decreased to 5° and 2°, and the kyphosis decreased to 11°. Radiographs at 4-year follow-up (**g**, **h**) showed good spinal balance with coronal segmental scoliosis of 9° and 2° and segmental kyphosis of 5°

All the patients were performed with posterior hemivertebra resection and bilateral transpedicular instrumentation. Somatosensory evoked potential (SEP) and motor evoked potential (MEP) were used intraoperatively. The patients were positioned prone on a radiolucent operation table after general anesthesia. For lumbar hemivertebra, one upper and one lower vertebrae were needed to be fused (Fig. 1). For thoracic hemivertebrae, two upper and two lower vertebrae were needed to be fused. Needles were inserted into the pedicles of the hemivertebrae and the adjacent vertebrae to identify hemivertebra by intraoperative fluoroscopy. After tapping and confirmed by gentle palpation using a sounder probe, screws with 3.5- to 5-mm diameter and 20- to 30-mm length were inserted into the pedicles. The posterior elements of the hemivertebra, including the lamina, upper and lower facets, and the transverse process, were removed to expose the pedicle and the nerve roots above and below. The pedicles, vertebra body, and the upper and lower disks were exposed and removed until the bleeding bone in the osteotomies. In the thoracic spine, the rib head and the proximal part of the surplus rib on the convex side were exposed and resected. In patients with contralateral bar and rib synostosis, the bar was cut and the synostosed rib heads were removed. A mesh cage filled with autologous bone graft was needed for patients with large hemivertebra or with obvious kyphosis to correct kyphosis deformity and provide instant postoperative stability. After compression to close the osteotomy gap, examination was needed to make sure that the exiting nerve roots and the dura were not impinged.

All patients were mobilized after the drainage tube was pulled out, and plastic braces were recommended to wear for at least 3 months. All the patients were required for regular follow-up after 3, 6, and 12 months of the surgery and every year afterwards in the follow-up.

### Statistical methods

Statistical analyses were performed using SPSS statistics software 19.0 (IBM, NY). Radiographic parameters were presented as mean ± standard deviation. Paired *t* tests were used to assess the differences of radiographic parameters between pre- and postoperative and postoperative and the last follow-up. And a *P* value of less than 0.05 was regarded as a significant difference.

## Results

A total of 43 hemivertebrae were resected in 39 cases, including one hemivertebra in 35 cases and two hemivertebra in 4 cases. The mean operative time was 189.8 min (90–415 min). Mesh cages were used in four cases to fill the large osteotomy gap after hemivertebra resection and to correct the segmental kyphosis. The mean estimated intraoperative blood loss was 306.6 ml (100–700 ml). The average follow-up time was 5.4 years (3–11 years).

The correction results of the coronal and sagittal planes were summarized in Table 1. The correction rate of coronal segmental scoliosis was 83.6 %, from 38.4° pre-operation to 6.3° post-operation. There was a loss of 3.2° in the last follow-up. The correction rate of sagittal segmental kyphosis was 81.9 %, from 17.7° pre-operation to 2.9° post-operation. There was a loss of 3.2° in the last follow-up. The spontaneous correction rates of the compensatory cranial curve and compensatory caudal curve were 65.7 and 66.9 %, with a loss of 0.9° and 0.6° at the last follow-up, respectively. Significant statistical differences were found in the coronal segmental and main curves and cranial and caudal compensatory curves between pre- and postoperative, but no significant statistical differences were found between postoperative and at the last follow-up. Significant statistical differences were found in pre- and postoperative thoracolumbar kyphosis and lumbar lordosis between pre- and postoperative and postoperative and at the last follow-up, but no significant statistical differences were found in thoracolumbar kyphosis between postoperative and at the last follow-up. No significant statistical differences were found in CB and SB between pre- and postoperative and postoperative and at the last follow-up.

**Table 1:** Summary of radiographic parameters preoperatively, postoperatively, and at the last follow-up in 39 cases

	Pre-operation	Post-operation	Final follow-up
Cobb’s angle of segmental main curve (°)	39.3 ± 12.2	6.3 ± 7.2^a^	9.5 ± 10.4
Compensatory cranial curve (°)	13.9 ± 9.8	4.6 ± 6.2^a^	5.5 ± 7.6
Compensatory caudal curve (°)	13.9 ± 10.7	4.6 ± 6.0^a^	5.2 ± 8.4
Cobb’s angle of segmental kyphosis (°)	17.7 ± 17.0	3.2 ± 2.8^a^	6.4 ± 10.7
Apical vertebral translation (mm)	23.9 ± 9.6	7.0 ± 10.2^a^	6.4 ± 10.7
Coronal balance (mm)	13.7 ± 11.1	11.8 ± 13.4	7.8 ± 9.7
Sagittal balance (mm)	−3.4 ± 28.6	4.2 ± 32.1	1.7 ± 23.0
Thoracic kyphosis (°)	22.9 ± 15.8	22.1 ± 11.4	26.6 ± 15.2
Thoracolumbar kyphosis (°)	12.8 ± 14.3	4.8 ± 7.7^a^	6.9 ± 12.9
Lumbar lordosis (°)	−46.2 ± 23.8	−36.9 ± 12.9^a^	−44.1 ± 12.9^b^

^a^Paired *t* test between preoperative and postoperative. Significant differences were considered for *P* value less than 0.05

^b^Paired *t* test between postoperative and final follow-up. Significant differences were considered for *P* value less than 0.05

**Fig. 2:**
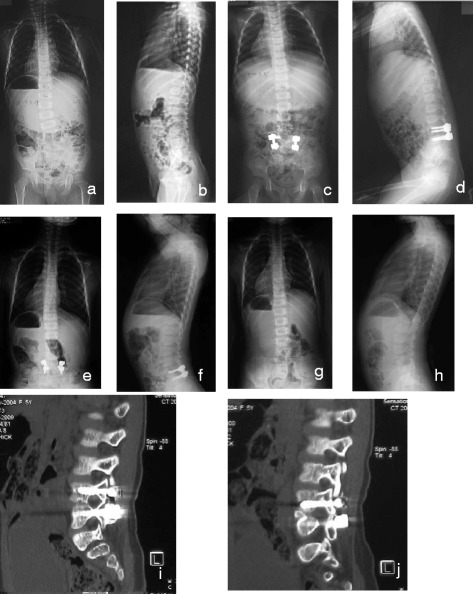
An infant with congenital scoliosis. Preoperative radiographs (**a**, **b**) showed fully segmented hemivertebra of L5/6 with the Cobb angle of 27°. Postoperative radiographs (**c**, **d**) showed excellent correction by L5/6 hemivertebra resection with the Cobb angle of 2°. Radiographs at 3 years follow-up (**e**, **f**) showed excellent correction with the Cobb angle of 4°. CT scan (**i**, **j**) showed solid fusion at the fused segments and L5 right pedicle screw dislodgement. And implants were removed (**g**, **h**)

There were no major vascular or neurological complications in this study. There were no pseudarthrosis in any of the patients at the last follow-up, and no patients showed symptoms of spinal stenosis in the follow-up. Three patients suffered from complications and two patients received anticipated surgery. There was one case of pedicle fracture at right L3 pedicle, and revision surgery was performed to replace the misplaced screw with a hook. One patient had one rod brakeage 6 months after the surgery. No revision surgery was done and no rod migration or scoliosis aggravation in the follow-up. One case had progressive kyphosis after the operation and developed significant kyphosis 7 years later, and revision surgery was indicated. Besides these, two patients had additional implant removal surgery at 4 and 7 years’ follow-up. Obvious instrumented pedicle elongation and implant migration were found in the patients. CT scan showed solid fusion in the posterior elements and interbody segments, and implant removals were performed after the confirmation of fusion in the operation (Fig. 2).

## Discussion

As the most frequent cause of congenital scoliosis, most untreated fully segmented and semi-segmented non-incarcerated hemivertebra will progress and create a wedge-shaped deformity during the spinal growth period [1, 4]. Delayed surgery has to include the rigid structural compensatory curve for fusion and has higher neurologic risk. A comparative study, conducted by Chang et al., showed that compared with patients treated after 6 years old, patients treated before the age of 6 years had significantly better deformity correction and did not cause a negative effect on the growth of the vertebral body or spinal canal [27]. Therefore, in order to achieve better correction results and better spinal mobilization in the future, earlier surgical intervention is indicated for most congenital scoliosis patients.

Posterior hemivertebra resection with transpedicular instrumentation was first introduced by Jürgen Harms in 1991. Compared with one-stage or two-stage AP hemivertebra resection, the advantage of posterior hemivertebra resection, including good correction results in the coronal and sagittal planes, less invasive, lower complication rate, and shorter postoperative recovery period, makes it more suitable for young children to deal with this kind of deformity [26]. Ruf and Harms first reported posterior hemivertebra resection with transpedicular instrumentation. In 2003, they reported 28 cases of mean age 3.3 years old with a mean follow-up of 3.5 years, including 25 cases treated with bisegmental fusion [14]. The mean correction rate of the main curve was 71.1 %, and the correction rate of segmental kyphosis was 63 %. The spontaneous correction rates of cranial and caudal compensatory were 78 and 65 %, respectively. In 2009, they reported 41 cases of mean age 3.4 years old with a mean follow-up of 6.2 years [17]. In patients without bar formation, bisegmental fusion was chosen and they had achieved similar results as before. But in patients with bar formation, the correction rates of the main curve and segmental kyphosis were 66.7 and 62.5 %, respectively, and the correction rates of the cranial and caudal compensatory curves were 74.8 and 75.1 %, respectively, which were less than those of patients without bar formation. With limitation of instrumentation, some of their cases were performed with wire or screw. Zhang et al. conducted a study of 56 patients of mean age 9.9 years old with a mean follow-up of 32.9 months, and 11 cases chose bisegmental fusion [18]. There was a mean improvement of 72.9 % in the segmental scoliosis and 70 % in segmental kyphosis. In our study, all the cases were treated with posterior-only hemivertebra resection with transpedicular instrumentation with mean age of 3.4 years old for at least 5.4 years’ follow-up, and 22 cases chose bisegmental fusion. The correction results of segmental scoliosis and segmental kyphosis are 83.6 and 81.9 %, respectively, which were similar to previous reports.

Although earlier surgeries were advocated by most surgeons, the appropriate age for children having surgery is still controversial. The earlier the surgery is, the milder the deformity is, the shorter the fusion segment is, and the better result they will achieve. Pedicle screws have to pass through the neurocentral cartilage (NCC), which is responsible for the growth of the pedicles and the posterior vertebral body, and NCC starts to fuse in the lumbar segments around the age of 3 years and in thoracic segments around the age of 5 years [28]. Pedicle screw fixation on children younger than 5 years old has always been on debate. Until now, there have been no reports on iatrogenic neurogenic claudication caused by spinal stenosis due to implants. Ruf and Harms reported 41 cases with posterior hemivertebra resection and the youngest child treated was 1 year and 4 months [17]. No spinal stenosis or neurologic deficits was found in the follow-up. Crostelli et al. reported 15 cases with posterior lumbar or thoracolumbar hemivertebra with mean follow-up of 40 months, and the youngest patient treated by this procedure was 18 months old at the time of surgery [21]. No major complications emerged and only one pedicle fracture occurred. In our study, the youngest children performed were 1 year and 6 months and the longest follow-up time was 11 years. No neurological or vascular impairment due to surgery and implants was found in the follow-up. Considering the safety of anesthesia and children’s compliance with postoperative nursing care, we believe that it is appropriate for congenital scoliosis patients to perform posterior hemivertebra resection after 2 years old in our clinical practice.

Young age has always been a big challenge for patients to have posterior hemivertebra resection. In the early clinical reports of posterior hemivertebra resection with transpedicular instrumentation, Ruf and Harms reported 28.5 % of 28 cases with mean age of 3 years and 4 months treated by posterior hemivertebra resection suffered from complications, including two pedicle fractures, three instrumentation failures, two additional surgeries for curve progression, and one infection [13]. In their later reports, 29.3 % of 41 cases younger than 6 years old with a mean follow-up of 6.2 years suffered from complications, including 3 implant failure, 3 convex pedicle fracture, 2 additional operations for new deformity in group 1 without bar formation and 1 revision for hematoma, 1 infection, and 2 additional surgery for new deformity in group 2 with bar formation [17]. In 2011, Zhang et al. reported a 10.8 % complication rate in 56 cases during a mean follow-up of 32.9 months, including one delayed wound healing, two pedicle fractures, two rod breakages, and one additional surgery for proximal junction kyphosis [18]. In our study, there were three complications, including one pedicle fracture, one rod breakage, and one additional surgery for new deformity. Besides, two additional implant removal were performed in this study because of elongation and implant migration in the follow-up.

The cause of complications in congenital scoliosis may include limitation of the operative procedure itself, improper operation during the surgery, improper posterior fusion, incomplete hemivertebra resection, or improper fusion level. Strategies should be taken to deal with this problem [27]. Firstly, location of hemivertebra should be confirmed and pedicle tunnel should be prepared correctly before screw insertion. Preoperative spine CT scan parallel to the pedicle and 3D reconstruction should be done to evaluate the morphology of pedicles and help to choose proper instrumentation. Secondly, the whole hemivertebra, including the cartilage, facet joints, endplates, and disks above and below should be completely removed until the bleeding bone. Besides, the transverse process or the rib adjacent to hemivertebra, contralateral bar, or rib synostosis in the fusion segments should also be resected before compression. Thirdly, mesh cage is needed for patients with large hemivertebra or obvious kyphosis to reconstruct the anterior column and increase the immediate postoperative stability. Fourthly, posterior elements, especially adjacent paraspinal musculature and ligaments, should be protected during the operation to prevent junctional kyphosis. The last is that tutorization in brace after surgery for at least 3 months and regular clinic follow-up after operation. Protection in brace for enough time may be helpful for better fusion results.

This study has some limitations. This is a retrospectively reviewed study with small number of patients included; more patients are needed in the future. The second limitation is that the patients included in the study are very young and far from mature bone and continuous follow-up are needed in the future. The third limitation is that this study does not contain results about life quality in the follow-up. Further trials about life quality, spinal mobilization, and pain are needed in the future.

## Conclusions

Posterior hemivertebra resection with transpedicular instrumentation is a safe and effective procedure in treatment of congenital scoliosis cases, even for children under 5 years old. Earlier surgeries can not only achieve better correction results but also help to prevent secondary curve progression and save more motion segments. However, complications still exist in peri-operative period and follow-up. As most of the patients in our study are still young, continuous follow-up are still needed in the further.

## Abbreviations

AP: anterior–posterior
CB: coronal balance
L: lateral
SB: sagittal balance
